# Management trends and practices in ischial tuberosity avulsion fractures: a cross-sectional study among hip surgeons in the UK, surgical technique and literature review

**DOI:** 10.1308/rcsann.2025.0008

**Published:** 2025-03-25

**Authors:** NA Shaharudin, HA Al Hussainy, O Shannak, G Mundy

**Affiliations:** Northampton General Hospital NHS Trust, UK

**Keywords:** ITAF, Ischial tuberosity, Surgical management, Cross-sectional-study, Case report, Literature review

## Abstract

**Background:**

Ischial tuberosity avulsion fracture (ITAF) is a rare injury affecting predominantly adolescent athletes yet lacks standardised management protocols. This study aims to investigate the diverse management preferences among hip surgeons regarding ITAF and share our preferred surgical technique and management.

**Methods:**

In a cross-sectional study, 237 British Hip Society members were surveyed regarding various aspects of ITAF management, including preferences for operative versus non-operative approaches, surgical techniques and postoperative rehabilitation regimens. Sixty-two surgeons responded, yielding a 26% response rate.

**Results:**

Thirty-six surgeons (58.1%) favoured conservative treatment, while 26 (41.9%) preferred surgery based on the degree of displacement. Among those advocating for surgery, 16 (61.5%) deemed displacement ≥20mm as significant, with 5 (19.2%) considering ≥15mm significant and another 19.2% regarding any displacement as significant. Prone theatre positioning was overwhelmingly preferred by 96.2%, with a majority (65.4%) favouring the transverse gluteal crease approach. Postoperatively, 11.5% preferred immediate full weight bearing, while 88.5% opted for six weeks of non-weight-bearing following surgery.

Among conservative management advocates, 29% allowed unrestricted weight-bearing post-injury, 11.3% preferred weight-bearing until further review and 59.7% opted for partial weight-bearing for at least six weeks.

**Conclusions:**

This study highlights the absence of a consensus on ITAF management. We present our preferred approach through a case analysis involving an ITAF patient treated at our department to enhance understanding of this rare injury and potentially improve management strategies.

## Introduction

Ischial tuberosity avulsion fracture (ITAF) is a rare but impactful injury, typically occurring due to an abrupt overload on the hamstring muscles via forceful flexion of the hip joint with the knee extended.^1,2^ This traumatic event often presents with pain in the proximal posterior thigh region and localised swelling. Adolescents engaged in high-impact competitive sports, notably football and gymnastics,^3^ are particularly susceptible to ITAF due to the vulnerability of their growth plates to trauma.^4,5^

The consequences of delayed diagnosis of ITAF can be severe, resulting in chronic pain, compromised athletic performance and the potential need for extensive surgical interventions.^6^ Despite its clinical significance, the management of ITAF remains without a standardised consensus. Clinicians often consider the degree of fracture displacement and involvement of the sciatic nerve as factors in determining whether conservative or surgical interventions are warranted for affected patients.^2,7,8^

This study explores myriad aspects of ITAF management practised by hip surgeons across the UK. We aim to contribute to the discussions by presenting our insights through a case analysis involving an ITAF patient treated at our department. By exploring the diverse approaches and preferences, we seek to enhance understanding of this rare injury and potentially improve management strategies.

## Methods

Between April and October 2023, a cross-sectional study was conducted using an online smart questionnaire created through Google Forms.

A comprehensive list of consultant surgeons was obtained from the British Hip Society (BHS), identifying 237 registered members.

The questionnaire, tailored for this study, was distributed via email. A total of 62 responses were received, resulting in a response rate of 26.2%; 41 (66.1%) of the respondents specialised in arthroplasty, 10 (16.1%) were paediatric surgeons, 5 (8.1%) specialised in pelvic surgery and 6 (9.7%) specialised in sports medicine.

The questionnaire was designed to gather information on the frequency with which surgeons encounter actual ITAF cases, the proportion of cases requiring surgical stabilisation and the influence of fracture displacement on treatment decisions. Respondents were presented with options to assess displacement, including any displacement, >15mm, >20mm or non-operative management. Subsequent sections of the questionnaire were tailored according to the management approach selected by the respondents.

For respondents favouring surgical management, five additional questions were posed covering patient positioning, preferred surgical approach, fixation methods, sciatic nerve exposure and post-operative rehabilitation protocols.

Conversely, respondents opting for conservative management were presented with a different set of questions focusing on their preferred conservative treatment regimen.

All respondents were then directed to a third section of the survey, where questions addressed their approach to assessing functionality at follow-up and any additional comments regarding ITAF.

Data collected were tabulated and analysed using Microsoft Excel software to identify trends and patterns in the management preferences and practices of hip surgeons regarding ITAF.

## Results

Of the 62 respondents, the average frequency of encountering ITAF patients per surgeon per year was 0.91 (SD±1.1). Among these cases, an average of 0.18 (SD±0.6) required surgical stabilisation per surgeon per year; 36 (58%) surgeons favoured conservative management, while 26 (42%) surgeons based their treatment decisions on the degree of displacement.

**Figure 1:**
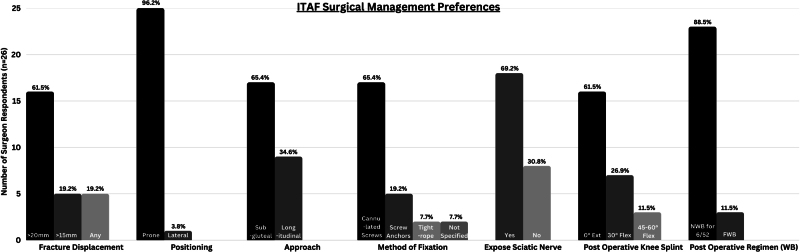
Graph illustrating detailed different preferences in the surgical management of ITAF according to the BHS member questionnaire responses. See text. BHS = British Hip Society; ITAF = Ischial tuberosity avulsion fracture.

Among the 26 respondents who based their decision on displacement, 16 surgeons deemed ≥20mm displacement as significant, 5 deemed ≥15mm displacement significant and 5 surgeons considered any degree of displacement to be significant for a surgical management (Figure 1).

For the 26 surgeons favouring surgical management, 25 preferred prone positioning and one preferred lateral positioning; 17 surgeons favoured the subgluteal approach at the gluteal crease, while the remaining 9 favoured a longitudinal incision. Seventeen surgeon preferred cannulated screws, five chose screw anchors, two opted for tightrope and two surgeons did not specify a fixation method. Eighteen would expose the sciatic nerve, while eight would not. Three surgeons allowed immediate full weight bearing, and 23 preferred non-weight bearing for six weeks followed by reassessment. Sixteen respondents preferred 0-degree extension at the knee joint, seven chose 30-degree flexion, and three opted for 45–60-degree flexion (Figure 1).

Among the 36 who preferred conservative management, 12 allowed unrestricted weight-bearing, 20 recommended partial weight-bearing for six weeks and 4 advised against weight-bearing for six weeks following the injury. In the context of knee immobilisation regimes, 2 surgeons preferred maintaining the knee locked in extension, while 27 advocated for freely mobilising the knee. Additionally, five surgeons suggested keeping the knee at a 30-degree flexion angle, and two proposed maintaining it at a 60-degree flexion angle.

Regarding functionality assessment at follow-up and out of the total of 62 respondents, 40 surgeons did not use a scoring system. Among those who did, the Perth Hamstring Assessment Tool (PHAT) was mentioned by seven, Harris Hip Score by six, Oxford Hip Score by four, Lower Extremity Functional Scale by three, and one respondent for each of EQ-5D and the Non-Arthroplasty Hip Registry.

## Case report

A 14-year-old adolescent presented to our institution after sustaining a football injury. The injury occurred when he fell forwards, landing on a hyperflexed hip with the knee in full extension. Following the incident, he experienced pain and limping, prompting his visit to the hospital. He had no known co-morbidities. Upon approval from the local ethics committee, both the patient and the legal guardian agreed to participate in this study and provided consent for the publication of the associated clinical images.

**Figure 2:**
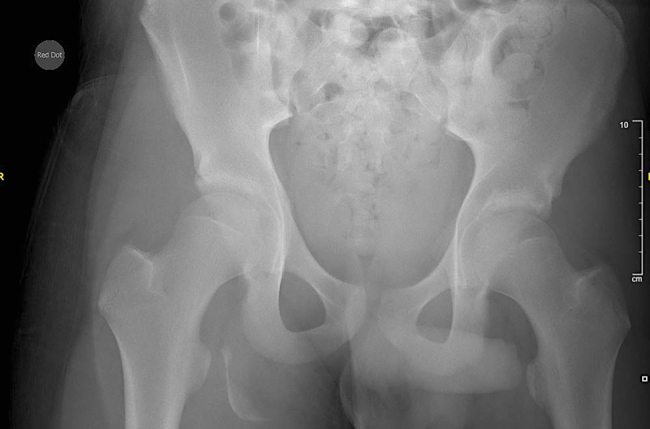
Preoperative anteroposterior pelvic radiograph showing the displaced right ITAF. ITAF = Ischial tuberosity avulsion fracture.

Initial imaging, including pelvic x-rays and magnetic resonance imaging (MRI) scans, revealed a significantly displaced right apophyseal ITAF at 19mm with the fragment measuring 48×24×12mm (Figure 2). Despite the fracture, there were no symptoms of sciatic nerve injury, but the patient exhibited reduced active and passive range of motion and profound tenderness in the gluteal region.

Considering the size and the degree of displacement of fracture, and the patient's desire for functional recovery, a decision was made to proceed with open reduction internal fixation surgery.

## Surgical technique

Following appropriate consent, the patient underwent general anaesthesia and was positioned prone on a radiolucent operating table. Local guidelines were adhered for antibiotic protocol, surgical draping and aseptic skin preparation. The surgical field was sealed preoperatively using an adhesive ISODrap U-drape (Mictotek Medical Inc., Columbus, Mississippi, USA) to protect it from faecal contamination.

**Figure 3:**
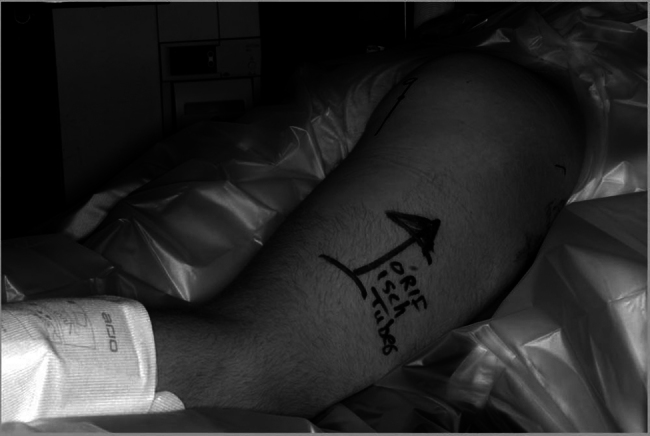
The patient positioned prone on theatre table. Notice the skin marking for the surgical incision. The small gluteal circle represents the skin landmark for the ischial tuberosity as identified with the image intensifier.

**Figure 4:**
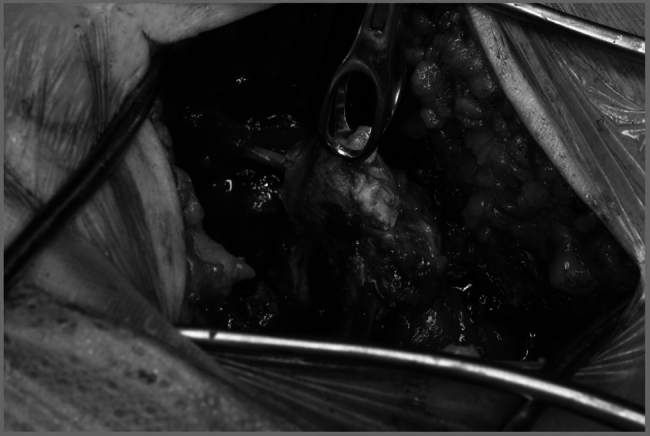
The displaced ITAF freed and lifted with a tissue holding forceps along with the hamstring muscles attached ready to be stabilised to the right ischial bone of the pelvis. ITAF = Ischial tuberosity avulsion fracture.

Under x-ray control, a pre-marked longitudinal surgical incision was made, centred over the ischial tuberosity (Figure 3). The gluteal fascia was divided, and the gluteus maximus muscle was retracted superiorly to expose the fractured fragment of the ischial tuberosity (Figure 4). Throughout the procedure, great care was taken to protect the sciatic nerve.

**Figure 5:**
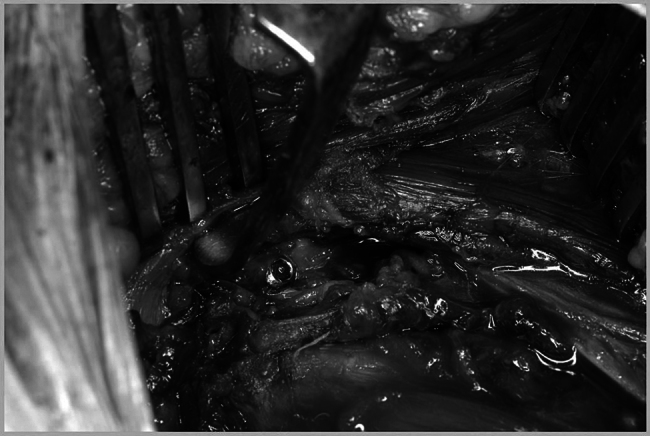
Cannulated screw inserted in place.

The fracture was manipulated and temporarily reduced using two 2mm K-wires. Subsequently, the fracture was stabilised rigidly using three partially threaded cannulated 4mm screws with washers in three different planes (Figure 5). This fixation method ensured good anatomical reduction and stability of the osteosynthesis. Confirmation of the reduction was done using intraoperative imaging, including antero-posterior, inlet, outlet and Judet views.

The surgical wound was closed in layers, with the skin being closed using a subcuticular suture technique and sealed with surgical glue to minimise the risk of faecal contamination.

**Figure 6:**
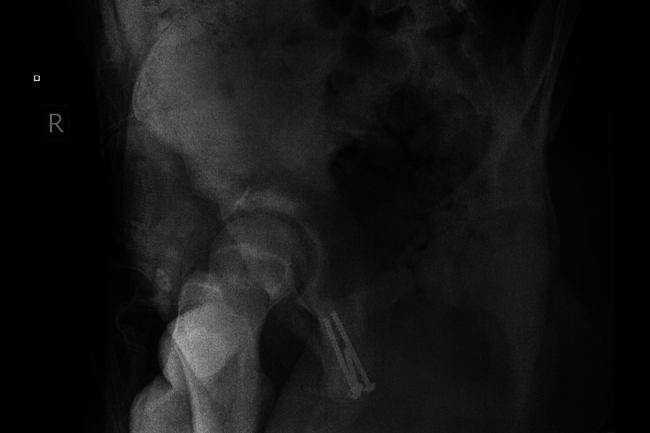
Judet view radiograph of the right hip one month postoperatively showing the fixation in place.

Postoperatively, the patient's leg was placed in a hinged knee brace locked at 50 degrees knee flexion for three weeks to prevent hyperextension at the hip joint. Radiographic assessment was carried out at four weeks follow-up (Figure 6). Subsequently, the knee hinge was adjusted to 30 degrees for another three weeks before being discarded at six weeks postoperatively. The patient was instructed to avoid hip flexion of more than 60 degrees. Non-weight-bearing mobilisation was allowed immediately for six weeks under physiotherapy supervision. At three months postoperatively, the patient was encouraged to resume sporting activities.

At 18 months postoperatively, our patient reported full functional recovery. Functionality was assessed using the modified Harris Hip Score (mHHS) and the PHAT. The patient achieved a full score of 100 on the mHHS and 85 out of 100 on the PHAT, with the minor deduction due to age-related restrictions. The patient has since returned to his pre-injury level of sport without any limitation or pain, scoring zero out of ten on the visual analogue pain score at rest, during daily living and sport activities.

## Discussion

The management of ITAF lacks consensus despite numerous published studies. A retrospective literature review yielded 82 relevant articles on ITAF, with 39 studies meeting inclusion criteria for analysis. The summary of the key findings is presented in Table 1. While many authors advocate for surgical intervention when appropriate,^1,6,9–31^ there is a considerable variability in defining the threshold for surgical management.

**Table 1:** Summary of the literature review.

Author, Year	*n*	ITAF key management points
Vadhera *et al*,^31^ 2022	90*	Good outcome for both operative and non-operative treatments. Return to sport in 94.7%, and 32.7% after operative and non-operative treatment respectively. Complication rate is higher in conservative treatment
Best *et al*,^18^ 2021	11^†^	Recommends surgery for >15mm displacement
Nauta *et al*,^8^ 2020	90*	Good outcome for both operative and non-operative treatments. Treatment option based on displacement. Insufficient data on timing of surgery
Calderazzi *et al*,^32^ 2018	n/a^‡^	Operative treatment preferred for major displacement, larger fragments, quicker recovery and to reduce pseudoarthrosis risk. Non-operative treatment preferred for minimally displaced fractures, with risks of non-union and potential need for delayed surgery
Liu *et al*,^7^ 2018	n/a^‡^	No consensus; conservative treatment is the most common. ITAF often misdiagnosed. Factors to consider: fracture size, displacement and the patient's functional needs
Eberbach *et al*,^30^ 2017	177*	Outcome of surgery is better in high functional demands with displacement >15mm
Sinikumpu *et al*,^28^ 2017	11^†^	More than 80% of patients treated surgically returned to preinjury sport levels
Schuett *et al*,^2^ 2015	24^†^	Successfully managed 97% of patients conservatively. AIIS and ischial tuberosity fractures—increased risk of future pain and non-unions
Spencer-Gardner *et al*,^33^ 2015	10^†^	Surgery in ITAF malunion/non-union improved symptoms, function, and hamstring strength
Schoensee *et al*,^17^ 2014	3^†^	Successfully managed delayed ITAF union with percutaneous needle fenestration
Ferlic *et al*,^1^ 2013	13^†^	Recommended conservative treatment for <15mm and surgery for active patients >15mm displacement
Raissaki *et al*,^5^ 2007	n/a^‡^	Focuses mainly on the radiological aspect adolescent sport injury
Gidwani *et al*,^6^ 2007	12^†^	Recommended surgery for acute displaced cases
Rossi *et al*,^3^ 2001	109^†^	ITAF common in football and gymnastics. Aetiology: sudden, forceful musculotendinous contractions. Diagnosis: determined by plain radiographs
Kujala *et al*,^4^ 1997	21^†^	Recommended conservative treatment

AIIS = anterior inferior iliac spine; ITAF = Ischial tuberosity avulsion fracture; *n* = number of patients in each cohort; WB = weight bearing.

*Systematic review.

^†^Retrospective study.

^‡^Literature review.

Liu *et al* (2018) highlighted that the decision to manage ITAF surgically often hinges on the degree of fracture displacement, especially in the absence of sciatic nerve injury.^7^ However, the specific threshold with regards to the degree of displacement that warrants surgical management differs among authors. Fracture displacements less than 15mm are commonly managed non-operatively,^4,7,8,18,34,35^ while displacements exceeding 20mm often prompt surgical intervention, even in the absence of sciatic nerve involvement.^15,21,26,36^ The presence of sciatic nerve injury was often an indication for emergency surgery.^7,15^

Various authors have advocated for conservative treatment in cases of ITAF, especially when the displacement is not significant, typically defined as not exceeding 20mm according to various sources. ^4,7,8,24,30–32,34–37^ This conservative approach typically involves a period of bed rest ranging from two to six weeks, alongside modified restricted activity and rehabilitation through physiotherapy.

Surgeons who support conservative management often cite several reasons for their preference over surgical intervention:

Limited anatomical familiarity: surgeons may lack experience with the anatomy of ITAF due to its infrequent nature and the prone positioning required – inverting their anatomical perspective;
Iatrogenic risks: surgery near the ischial tuberosity carries inherent risks to surrounding structures including nerves;
Hygiene concerns: surgeons may express concerns about the proximity of the surgical site to potential sources of contamination, such as faecal matter, which could lead to post-operative complications.

Our survey revealed 58.1% prefer conservative approaches for managing ITAF, likely due to these aforementioned reasons, among others.

For improved cosmetic outcomes, the transverse incision surgical approach has been widely favoured in the literature.^6,7,14,21,38^ This approach involves making an incision along the crease or inferiorly parallel to the gluteal crease in a subgluteal fashion, a preference also reflected in our survey findings. Some surgeons opt for a combination of incisions, including longitudinal and subgluteal approaches,^10–12,15,22^ while Watts *et al* in 2014 favoured the Kocher–Langenbeck approach.^6,25^ All of these approaches have demonstrated favourable outcomes.

In our clinical experience, we chose a direct longitudinal approach extending from the ischial tuberosity inferiorly and downwards. This approach offers advantages in terms of extendibility, flexibility and optimal visualisation of the hamstrings and the fractured fragment, while also providing optimal protection of the surrounding structures at risk. Additionally, this approach facilitates the identification of the posterior cutaneous nerve of the thigh, potentially reducing the risk of injury.

A grey area lies in the 15–20mm displacement range, where outcomes can be favourable with both surgical and conservative approaches.^1,18,30,32,34,38^ Patient factors such as expectations, functional demands (especially in athletic individuals) and willingness to comply with postoperative rehabilitation play crucial roles in treatment decision-making. In our case study, the patient's high functional demand and aspirations for competitive sports influenced the decision for surgical management.

The surgical approach varies among practitioners, with prone positioning being the most popular choice,^10–15,18,19,21,22,25,27,28^ although lateral positioning has also demonstrated success.^1^ Misdiagnosis of ITAF injuries is also common, leading to delayed treatment and potential complications such as chronic pain or sciatic nerve palsy.^6,31,38^ Some authors advocate for pelvic MRI scans to avoid missed diagnoses, particularly in athletic adolescents presenting with gluteal pain.^6,31^

A wide array of surgical stabilisation techniques, devices and principles have been employed in the literature. Various authors have utilised different screw types,^1,10,12–15,18,25^ and suture anchors, including the suture bridge technique.^11,18,19,27^ Some surgeons have preferred bone graft augmented fixation,^16,38^ partial resection with plate osteosynthesis,^36^ or reduction by osteotomy with plates and screws.^39^ This diversity in stabilisation methods was also reflected in our questionnaire, highlighting that there was no universally superior approach. In our case, we used cannulated screws with washers inserted at different planes. This stabilisation technique also reduces the amount of metalwork in situ, thereby minimising the risk of irritation to surrounding structures and discomfort to the patient.

After surgery, various orthotic devices have been used to aid in the recovery and rehabilitation process such as orthosis, the Snyder sling, hard frame hip and knee braces, and hip abduction braces among others.^10,11,19,27^ Typically, full weight bearing is permitted around six weeks following a surgical repair.

In terms of post-operative assessment, there is no standardised outcome measurement tool in both literature and our survey; 64.5% of respondents preferred not to even use a scoring system. Some studies, such as those by Eberbach *et al* in 2017, Best *et al* in 2021 and Vadhera *et al* in 2022, judged outcomes based on the absence of pain and the patient's ability to return to sports.^18,30,31^ Others, like Ferlic *et al* in 2013, Spencer-Gardner in 2015 and Nauta *et al* in 2020, preferred using the mHHS.^1,8,38^ Conversely, Best *et al* utilised the PHAT for assessing post-operative recovery.

In our case, we used both the mHHS and PHAT to evaluate the patient's functional outcome. However, both assessment tools have their limitations. The mHHS was not specifically designed to assess the hamstring, which is the primary muscle group involved in ITAF injuries. The PHAT, while focusing on hamstring functionality, includes aspects that may be irrelevant to the typical age group affected by ITAF, such as discomfort associated with driving.

Pre-existing scoring systems assessing hip and lower limb functionality have their place, but none are specifically tailored to address the unique characteristics and outcomes associated with ITAF.

Notable limitations of this study are the small sample size, and the choice of distributing questionnaires via email. Although convenient for accessing surgeons across various geographical locations, email-based studies are recognised as a lower-yield channel among healthcare professionals.^40^

There is an absence of non-operative rehabilitation regimes in literature. The lack of specific guidance from existing literature likely prompted surgeons to draw upon their own experiences, preferences and expertise when considering non-operative rehabilitation approaches for ITAF. This diversity of perspectives underscores the complexity of managing ITAF injuries and the importance of considering individual patient characteristics, preferences and needs when devising rehabilitation plans. Overall, the varied responses to the questionnaire emphasise the importance of comprehensive exploration and discussion of rehabilitation options, both surgical and non-surgical, in the management of ITAF injuries. Such discussions can help inform clinical decision-making and contribute to the development of evidence-based rehabilitation protocols tailored to the specific needs of ITAF patients.

The findings from the questionnaire, which assessed the frequency of encountering ITAF and their subsequent management by individual surgeons, highlighted the rarity of these injuries. On average, an orthopaedic surgeon would encounter approximately one patient with an ITAF injury per year and perform surgical stabilisation once every five years.

The survey conducted in conjunction with our literature review revealed a lack of consensus among surgeons in various aspect of ITAF management, strengthening the need for further research to establish evidence-based guidelines. Despite the limitations of our study, our findings contribute valuable insights into the current understanding and management of ITAF injuries.

## Conclusion

This study confirms the lack of consensus and standardised guidelines in the management of ITAF injuries. Treatment decisions remain heavily influenced by factors such as fracture displacement, patient preferences and surgeon expertise. Standardisation in outcome assessment tools is lacking, highlighting the need for further research to establish evidence-based guidelines for ITAF management. Our clinical experience demonstrates successful surgical management of ITAF, emphasising the importance of individualised treatment approaches tailored to patient-specific factors and functional goals.

## References

[1] Ferlic P, Sadoghi P, Singer G *et al.* Treatment for ischial tuberosity avulsion fractures in adolescent athletes. *Knee Surg Sports Traumatol Arthrosc* 2013; **22**: 893–897. 23793970 10.1007/s00167-013-2570-4

[2] Schuett D, Bomar J, Pennock A. Pelvic apophyseal avulsion fractures: a retrospective review of 228 cases. *J Pediatr Orthop* 2015; **35**: 617–623. 25321882 10.1097/BPO.0000000000000328

[3] Rossi F, Dragoni S. Acute avulsion fractures of the pelvis in adolescent competitive athletes: prevalence, location and sports distribution of 203 cases collected. *Skeletal Radiol* 2001; **30**: 127–131. 11357449 10.1007/s002560000319

[4] Kujala U, Orava S, Karpakka J *et al.* Ischial tuberosity apophysitis and avulsion among athletes. *Int J Sports Med* 1997; **18**: 149–155. 9081273 10.1055/s-2007-972611

[5] Raissaki M, Apostolaki E, Karantanas A. Imaging of sports injuries in children and adolescents. *Eur J Radiol* 2007; **62**: 86–96. 17306491 10.1016/j.ejrad.2007.01.012

[6] Gidwani S, Bircher M. Avulsion injuries of the hamstring origin—a series of 12 patients and management algorithm. *Ann R Coll Surg Engl* 2007; **89**: 394–399. 17535619 10.1308/003588407X183427 PMC1963576

[7] Liu H, Zhang Y, Rang M *et al.* Avulsion fractures of the ischial tuberosity: progress of injury, mechanism, clinical manifestations, imaging examination, diagnosis and differential diagnosis and treatment. *Med Sci Monit* 2018; **24**: 9406–9412. 30589058 10.12659/MSM.913799 PMC6322373

[8] Nauta H, van der Made A, Tol J *et al.* Satisfactory clinical outcome of operative and non-operative treatment of avulsion fracture of the hamstring origin with treatment selection based on extent of displacement: a systematic review. *Knee Surg Sports Traumatol Arthrosc* 2020; **29**: 1813–1821. 32809117 10.1007/s00167-020-06222-y PMC8126544

[9] Berry JM. Fracture of the tuberosity of the ischium due to muscular action. *J Am Med Assoc* 1912; **LIX**: 1450.

[10] Vasiliadis A, Tsioulas P, Alvanos D, Mpeletsiotis A. Avulsion fracture of the ischial tuberosity: a surgical dilemma. *Clin Case Rep* 2021; **9**: 1814–1815. 33768952 10.1002/ccr3.3854 PMC7981699

[11] Tetsunaga T, Endo H, Tetsunaga T *et al.* Avulsion fracture of the ischial tuberosity treated with the suture bridge technique: a case report. *BMC Musculoskelet Disord* 2019; **20**: 9. 30611250 10.1186/s12891-018-2377-z PMC6320617

[12] Ali A, Lewis A, Sarraf K. Surgical treatment of an ischial tuberosity avulsion fracture with delayed presentation. *J Clin Orthop Trauma* 2020; **11**: S4–S6. 31992908 10.1016/j.jcot.2019.07.010 PMC6978189

[13] Nakamatsu Y, Fukui T, Oe K *et al.* Surgically treated nonunion following ischial tuberosity avulsion fracture of a 14-year-old athlete. *Case Rep Orthop* 2020; **2020**: 8531648. 32607266 10.1155/2020/8531648 PMC7313159

[14] Hughes J, Stahl D. Ischial tuberosity avulsion fracture nonunions in the adolescent population treated with a posterior column screw: a case series of two patients. *J Orthop Surg* 2019; **27**: 230949901983902. 10.1177/2309499019839022 30939995

[15] Liu H, Li Q, Shi Y *et al.* Surgical treatment for acute ischial tuberosity avulsion fracture: a case report. *Medicine (Baltimore)* 2019; **98**: e15040. 30946345 10.1097/MD.0000000000015040 PMC6455987

[16] Stafford C, Colberg R, Nourse A. Chronic ischial tuberosity avulsion nonunion fracture treated with a platelet-rich plasma injection as a bone graft. *Regen Med* 2019; **14**: 353–358. 31070520 10.2217/rme-2018-0071

[17] Schoensee SK, Nilsson KJ. A novel approach to treatment for chronic avulsion fracture of the ischial tuberosity in three adolescent athletes: a case series. *Int J Sports Phys Ther* 2014; **9**: 974–990. 25540712 PMC4275201

[18] Best R, Meister A, Huth J *et al.* Surgical repair techniques, functional outcome, and return to sports after apophyseal avulsion fractures of the ischial tuberosity in adolescents. *Int Orthop* 2021; **45**: 1853–1861. 33963885 10.1007/s00264-021-04959-w PMC8266717

[19] Lutz P, Knörr M, Geyer S *et al.* Delayed proximal hamstring tendon repair after ischial tuberosity apophyseal fracture in a professional volleyball athlete: a case report. *BMC Musculoskelet Disord* 2021; **22**: 578. 34167498 10.1186/s12891-021-04468-2 PMC8223337

[20] Jorgensen J, Mølgaard C, Kristinsson J. Surgical fenestration and rehabilitation of a sports traumatic non-union ischial tuberosity fracture—case report. *Int J Surg Case Rep* 2018; **53**: 362–366. 30472632 10.1016/j.ijscr.2018.11.018 PMC6260376

[21] Kaneyama S, Yoshida K, Matsushima S *et al.* A surgical approach for an avulsion fracture of the ischial tuberosity: a case report. *J Orthop Trauma* 2006; **20**: 363–365. 16766942 10.1097/00005131-200605000-00012

[22] Saka G. A tuber ischium avulsion fracture treated with modified subgluteal approach: a case report. *Acta Orthop Traumatol Turc* 2012; **46**: 403–406. 23268827 10.3944/aott.2012.2650

[23] Spinner R, Atkinson J, Wenger D, Stuart M. Tardy sciatic nerve palsy following apophyseal avulsion fracture of the ischial tuberosity. *J Neurosurg* 1998; **89**: 819–821. 9817420 10.3171/jns.1998.89.5.0819

[24] Bolgla LA, Jones DL, Keskula DR, Duncan JB. Hip pain in a high school football player: a case report. *J Athl Train* 2001; **36**: 81–84. 12937518 PMC155407

[25] Watts C, Hartzler R, Cross W. Open reduction and percutaneous fixation of a rare hamstring avulsion fracture. *Case Rep* 2014; **2014**: bcr2014205256. 10.1136/bcr-2014-205256 PMC4180579 25257887

[26] Wootton JR, Cross M, Holt K. Avulsion of the ischial apophysis. The case for open reduction and internal fixation. *J Bone Joint Surg Br* 1990; **72-B**: 625–627. 10.1302/0301-620X.72B4.2380217 2380217

[27] Buckley P, Dodson C. Repair of a proximal hamstring rupture in a 14-year-old patient: a case report. *HSS J* 2018; **14**: 302–306. 30258337 10.1007/s11420-018-9620-x PMC6148575

[28] Sinikumpu J, Hetsroni I, Schilders E *et al.* Operative treatment of pelvic apophyseal avulsions in adolescent and young adult athletes: a follow-up study. *Eur J Orthop Surg Traumatol* 2017; **28**: 423–429. 29159479 10.1007/s00590-017-2074-x

[29] Dosani A, Giannoudis P, Waseem M *et al.* Unusual presentation of sciatica in a 14-year-old girl. *Injury* 2004; **35**: 1071–1072. 15351680 10.1016/S0020-1383(03)00104-9

[30] Eberbach H, Hohloch L, Feucht M *et al.* Operative versus conservative treatment of apophyseal avulsion fractures of the pelvis in the adolescents: a systematical review with meta-analysis of clinical outcome and return to sports. *BMC Musculoskelet Disord* 2017; **18**: 162. 28420360 10.1186/s12891-017-1527-z PMC5395880

[31] Vadhera A, Knapik D, Gursoy S *et al.* Avulsion fractures of the ischial tuberosity in the pediatric athlete: a systematic review and return to sport analysis. *J Pediatr Orthop B* 2022; **31**: 508–516. 35258027 10.1097/BPB.0000000000000968

[32] Calderazzi F, Nosenzo A, Galavotti C *et al.* Apophyseal avulsion fractures of the pelvis. A review. *Acta Biomed* 2018; **89**: 470–476. 30657114 10.23750/abm.v89i4.7632 PMC6502104

[33] Spencer-Gardner L, Bedi A, Stuart M *et al.* Ischiofemoral impingement and hamstring dysfunction as a potential pain generator after ischial tuberosity apophyseal fracture non-union/malunion. *Knee Surg Sports Traumatol Arthrosc* 2015; **25**: 55–61. 26429568 10.1007/s00167-015-3812-4

[34] Yang B, Yi S, Ahn Y *et al.* Ischial tuberosity avulsion stress fracture after short period of repetitive training. *Hip Pelvis* 2016; **28**: 187–190. 27777924 10.5371/hp.2016.28.3.187 PMC5067398

[35] Ceretti M, Di Renzo S. A new evaluation system for early and successful conservative treatment for acute ischial tuberosity avulsion. *Chin J Traumatol* 2013; **16**: 254–256. 23910684

[36] Rutetzki K, Palm H, Friemert B *et al.* Avulsion fractures of the ischial tuberosity and resulting ischiofemoral impingement—a case report with literature review. *Z Orthop Unfall* 2018; **157**: 308–315. 30481834 10.1055/a-0757-8494

[37] Biernacki J, Sugimoto D, D’Hemecourt P, Stracciolini A. Ischial tuberosity avulsion fracture in a young female ballet dancer. *J Dance Med Sci* 2018; **22**: 233–237. 30477613 10.12678/1089-313X.22.4.233

[38] Gidwani S, Jagiello J, Bircher M. Avulsion fracture of the ischial tuberosity in adolescents—an easily missed diagnosis. *BMJ* 2004; **329**: 99–100. 15242916 10.1136/bmj.329.7457.99 PMC449822

[39] Putman S, Rommens P. A case of hypertrophic ischial tuberosity non-union treated by closed wedge osteotomy and plate and screws fixation. *Arch Orthop Trauma Surg* 2013; **133**: 513–516. 23411936 10.1007/s00402-013-1695-8

[40] Meyer VM, Benjamens S, Moumni ME *et al.* Global overview of response rates in patient and health care professional surveys in surgery. *Ann Surg* 2022; **275**: e75–e81. 32649458 10.1097/SLA.0000000000004078 PMC8683255

